# Abiraterone-Associated Renal Damage in Patients with Advanced Prostate Cancer as a Risk Factor for Mortality and Chronic Kidney Disease

**DOI:** 10.3390/jcm14217559

**Published:** 2025-10-24

**Authors:** Marina Pujol-Pujol, Marta Rivero-Martínez, Javier Puente, Natalia Vidal, Marta Calvo, Cristina Riaza, Marta Álvarez-Nadal, Antolina Rodríguez-Moreno, Ana I. Sánchez-Fructuoso, Clara García-Carro

**Affiliations:** 1 Nephrology Department, San Carlos Clinical University Hospital, 28040 Madrid, Spain; 2 Biomedical Research Institute of San Carlos Clinical University Hospital, 28040 Madrid, Spain; 3 Medical Oncology Department, San Carlos Clinical University Hospital, 28040 Madrid, Spain; 4 Medicine Department, Complutense University, 28040 Madrid, Spain

**Keywords:** prostate cancer, abiraterone, acute kidney injury, chronic kidney disease, electrolyte imbalance, hypertension

## Abstract

**Background**: Prostate cancer is the most frequent malignancy in men, with an incidence of 21% of all diagnosed tumors in this population in Spain. Between 10 and 20% of patients with prostate cancer develop castration-resistant prostate cancer (CRPC). Abiraterone is widely used in CRPC and metastatic prostate cancer, but data on its renal safety are limited. **Methods**: We performed a single-center, retrospective observational study including patients with advanced prostate cancer who initiated abiraterone between January 2013 and July 2024 at Hospital Clínico San Carlos (Madrid, Spain). Patients were followed until December 2024. Renal events were defined as acute kidney injury (AKI), electrolyte imbalance, new onset or worsening hypertension (HTN), and/or volume overload. Risk factors and associations with mortality were analyzed using multivariate models. **Results**: Seventy-nine patients were included (mean age 76 ± 9.5 years; 70.9% CRPC; 89.9% metastatic disease). Median follow-up was 17 months. Renal events occurred in 63.3% of patients. Independent risk factors were metastatic disease (OR 13.335; 95% CI 1.418–124.444; *p* < 0.0235) and HTN (OR 3.336; 95% CI 1.091–10.206; *p* < 0.0347). Electrolyte imbalance occurred in 36.7% of patients. AKI developed in 30.4% of patients, of whom 50% progressed to chronic kidney disease. New/worsening HTN occurred in 25.5%, and volume overload occurred in 16.5%. Abiraterone discontinuation due to renal events was rare (4%). At the end of follow-up, 18.9% of patients had died. In a multivariate Cox analysis, AKI was identified as an independent predictor of mortality [HR 3.044 (95% CI 1.001–9.260); *p* = 0.05]. **Conclusions**: Renal events are common in patients treated with abiraterone, especially in those with metastatic disease and hypertension. AKI independently predicted mortality. Close monitoring of renal function and blood pressure is essential in this population.

## 1. Introduction

Prostate cancer is the most common malignancy among men in Spain, representing 21% of all diagnosed cancers in this population, according to the latest data from the Spanish Society of Medical Oncology (SEOM) [1]. Approximately 10–20% of patients develop castration-resistant prostate cancer (CRPC). CRPC is defined as three consecutive PSA increases (with at least two of them above 50% of the nadir), and a PSA level > 2 ng/mL or radiographic progression despite castrate serum testosterone levels, achieved through surgical castration or androgen deprivation therapy. CPRC can occur in both metastatic and non-metastatic settings [2].

The therapeutic landscape for prostate cancer includes surgery, radiotherapy, androgen deprivation therapy and novel androgen receptor inhibitors. Among these treatments, abiraterone is one of the available options. It is indicated in patients with newly diagnosed, high-risk, metastatic hormone-sensitive prostate cancer (mHSPC) in combination with androgen deprivation therapy (ADT). It can be used in patients with metastatic CRPC (mCRPC), who are asymptomatic or mildly symptomatic, after failure of androgen deprivation therapy in whom chemotherapy is not yet clinically indicated. Finally, it is indicated in patients with mCRPC whose disease has progressed on or after a docetaxel-based chemotherapy regimen. In addition, it is also prescribed in combination with ADT and radiotherapy in high-risk locally advanced prostate cancer [3]. Abiraterone is a selective inhibitor of 17α-hydroxylase/C17,20-lyase (CYP17), which catalyzes the conversion of pregnenolone and progesterone into precursors of testosterone, dehydroepiandrosterone, and androstenedione. It is therefore employed as an antiandrogenic hormonal antagonist. By inhibiting CYP17, it also reduces cortisol synthesis, leading to stimulation of adrenocorticotropic hormone (ACTH) and a shift in steroidogenesis toward aldosterone synthesis This process may lead to secondary hyperaldosteronism. To prevent ACTH stimulation, abiraterone is co-administered with prednisone or prednisolone.

Abiraterone acetate is a prodrug that is metabolized to abiraterone by esterases. Abiraterone undergoes exclusively hepatic metabolism; therefore, impaired liver function may increase its half-life and raise the risk of adverse events. It reaches peak plasma concentration approximately 2 h after administration, has a half-life of 15 h in healthy individuals, and exhibits a high protein binding rate (99.8%) [4]. Its primary route of elimination is via feces (88%), with minimal renal excretion (approximately 5%) [5].

Marbury et al. [6] demonstrated that the pharmacokinetics of abiraterone in patients with end-stage renal disease on hemodialysis were like those observed in healthy individuals, based on a small patient sample. They reported that the maximum concentration was slightly lower and the time to reach maximum concentration was approximately twice as long in the hemodialysis group compared to the healthy controls. Furthermore, no drug-related adverse events were reported, supporting the safety of abiraterone in this patient population.

Few published studies have evaluated the relationship between abiraterone use and kidney injury. Scailteux et al. [7] and Riekhof et al. [8] reported an increased incidence of acute kidney injury (AKI) in patients treated with abiraterone compared to enzalutamide, another drug used for advanced prostate cancer. Cases of AKI due to rhabdomyolysis induced by the combination of statins and abiraterone have also been described [9,10], attributed to the inhibition of the hepatic enzyme OATP1B1, which metabolizes statins, thereby causing muscle damage.

Based on our clinical experience, abiraterone is widely associated with renal adverse events, such as AKI, hypertension (HTN), electrolyte disturbances, and volume overload. Thus, we conducted a study aimed at determining the incidence of abiraterone-associated renal events, the independent risk factors for the development of these renal adverse events, and their relationship with mortality in a cohort of patients treated with abiraterone.

## 2. Materials and Methods

This was a single-center, retrospective observational study of patients with advanced prostate cancer who initiated abiraterone at San Carlos Clinical University Hospital (Madrid, Spain) between January 2013 and July 2024. Patients were followed until December 2024. Inclusion criteria: all patients aged >18 years with prostate cancer treated with abiraterone and older than 18 years old were eligible to be included in the study. The only exclusion criterion was age younger than 18 years old. All patients with prostate cancer treated with abiraterone during the study period were included in the study.

Data were collected from hospital pharmacy records and reviewed by nephrologists through medical records of all patients. All data were anonymized. Collected variables included demographic information, tumor stage, past medical history, baseline serum creatinine and other laboratory parameters, as well as survival outcomes.

A renal event was defined as the occurrence of acute kidney injury (AKI), electrolyte disturbances (hypokalemia and/or hypernatremia), new-onset or worsening hypertension, and/or volume overload.

Baseline creatinine was defined as the last available value before initiating abiraterone during the study period. AKI was defined according to Kidney Disease: Improving Global Outcomes (KDIGO) criteria, including severity stages (1, 2, and 3). A minimum follow-up period of 3 months was used to assess recovery of renal function or progression to chronic kidney disease (CKD) according to KDIGO guidelines.

For baseline sodium and potassium levels, the last available laboratory results before abiraterone initiation were collected. Hypokalemia was defined as potassium < 3.5 mmol/L and hypernatremia as sodium > 145 mmol/L. The lowest potassium and highest sodium levels during follow-up were recorded.

The diagnosis or worsening of hypertension was assessed through initiation of antihypertensive therapy during follow-up and review of medical record diagnoses (de novo hypertension, hypertension stage progression, hypertensive crises), using a blood pressure threshold of >140/90 mmHg as the diagnostic criterion. The same approach was used to define volume overload: initiation of diuretics and/or medical diagnoses of volume overload based on edema, weight gain or imaging findings.

Data on concomitant medications were unavailable for 49 patients, and baseline renal function data were missing for 4 patients; however, none of these patients experienced renal events during follow-up.

Data were analyzed using SPSS Statistics version 24.0 (IBM, Armonk, NY, USA). Results were expressed as frequencies for categorical variables and as mean ± standard deviation (SD) or median and interquartile range (IQR) for continuous variables. The Kolmogorov–Smirnov test was applied to determine whether quantitative variables were normally distributed. For comparison of means between two groups, Student’s t or Mann–Whitney U test was used, depending on the distribution of the variable. Univariate and multivariate logistic regression were used to identify risk factors and Cox survival analysis to identify risk factors associated with mortality. Model was adjusted by backward stepwise regression based on maximum likelihood estimators. Variables with a *p* < 0.05 in the univariate analysis, as well as those considered clinically relevant, were included. After excluding collinearity, the optimal subset of variables was selected through backward elimination. Possible interactions were assessed by introducing multiplicative terms. To investigate the risk factors for mortality, we performed a Kaplan–Meier survival analysis and a univariate Cox regression analysis. Variables with a *p* value < 0.15 in the univariate Cox analysis, as well as those considered clinically relevant (such as age), were included in the multivariate Cox regression model. The hazard ratio (HR) and the 95% confidence interval (CI) were reported. Two-sided *p*-values < 0.05 were statistically significant.

## 3. Results

During the study period, 79 patients were treated with abiraterone at our center; all accomplished the inclusion criteria, so all were included in the analysis. Baseline characteristics are shown in Table 1. The mean age was 76 years (SD ± 9.5). A total of 70.9% had CRPC, and 89.87% had metastatic disease. Median baseline creatinine was 0.9 mg/dL (IQR 0.8–1.05); 21.5% had known CKD, and 21.52% had experienced at least one prior AKI episode before starting abiraterone. The median follow-up was 17 months (IQR 11.25–25.27). In our institution, abiraterone is routinely administered with prednisone 10 mg as concomitant therapy. All patients included in the study had started prednisone at the time of abiraterone initiation.

Overall, 50 patients (63.29%) experienced a renal event during the study period. Of those, 30.38% presented with AKI, 36.71% presented with electrolyte imbalance, 25.52% presented with HTN and 16.45% presented with volume overload. Patients who developed renal events were similar in age, CRPC status, presence of diabetes mellitus, use of diuretics, baseline creatinine, previous CKD, and median sodium and potassium values at the time of abiraterone initiation. However, they were more likely to have metastases (98% vs. 75.86%; *p* < 0.0017) and presented more frequently history of previous AKI (30% vs. 6.89%; *p* < 0.011) (Table 1). In a multivariate analysis including baseline creatinine, history of HTN, previous AKI, and metastatic cancer, the history of HTN, and metastatic cancer were identified as independent risk factors for developing a renal adverse event (Table 2). Despite 50 patients developing renal adverse events, in only 2 patients abiraterone was stopped (4%).

A total of 24 patients (30.38%) developed AKI, with a median creatinine 1.65 mg/dL (IQR 1.40–2.65). Of these, 41.7% presented with AKI stage 1, 33.3% AKI stage 2, and 25% AKI stage 3; no patient required renal replacement therapy. Interestingly, half of these patients with AKI developed subsequent CKD: 66.67% stage 3A, 8.33% stage 3B, and 25% stage 4 at the end of follow-up of 17 months. Patients who developed AKI had a worse baseline creatinine [1 mg/dL (0.8–1.2) vs. 0.8 mg/dL (0.8–1); *p* = 0.0274], were more likely to have history of HTN (75% vs. 43.64%; *p* < 0.023), and had a prior history of AKI (33.3% vs. 14.55%; *p* < 0.0376) when compared to patients who did not develop AKI (Table 3). In a multivariate analysis including history of HTN, baseline creatinine and previous history of AKI, pre-existing HTN was identified as an independent risk factor for the development of AKI (Table 4). The median time from abiraterone initiation to AKI development was 18 months (IQR 7.25–43) (Table 5).

Regarding electrolyte imbalance, 29 patients (36.71%) experienced this complication during follow-up. Hypokalemia occurred in 25 patients (31.65%), with a median potassium of 3.2 mmol/L (IQR 3–3.3); 20% had potassium < 3 mmol/L, and only one patient developed severe hypokalemia < 2.5 mmol/L. Hypernatremia was observed in 9 patients (11.39%), with a median sodium level of 145.5 mmol/L (IQR 145–148); no patient had moderate-to-severe hypernatremia (>150 mmol/L). Median time to electrolyte imbalance was 10.5 months (IQR 4–27) after starting abiraterone.

New-onset or worsening HTN, defined as initiation or intensification of antihypertensive treatment, as mentioned before, occurred in 17 patients (25.52%), with a median onset of 10 months (IQR 2–32.5). Volume overload, defined as initiation or escalation of diuretic therapy and/or a medical diagnosis, occurred in 13 patients (16.45%). No patient developed acute pulmonary edema during follow-up. Median time to volume overload was 16 months (IQR 4.5–37) after abiraterone initiation.

**Figure 1:**
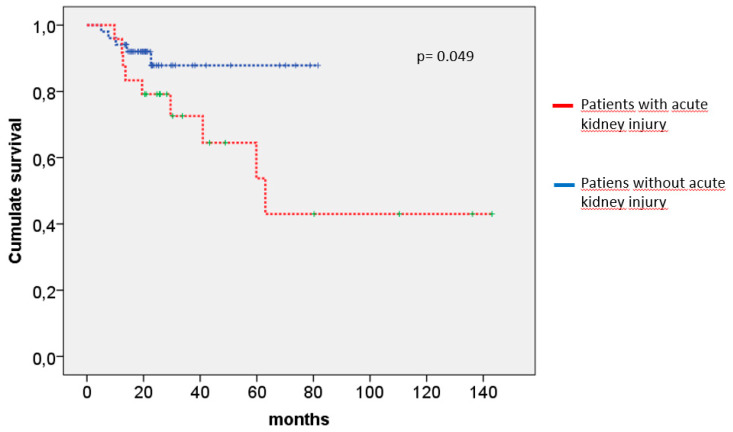
Kaplan–Meier survival curves comparing patients with and without acute kidney injury during abiraterone treatment. Patients who did not develop acute kidney injury had significantly longer survival compared to those who developed acute kidney injury.

At the end of follow-up, 15 patients (18.99%) had died, with a median survival of 13 months (IQR 9–29) after starting abiraterone. Patients who died were similar to survivors in terms of age, metastatic disease, CPRC, diabetes mellitus, CKD, and HTN before treatment, as well as the occurrence of renal events during follow-up. However, patients who experienced AKI during follow up presented a shortened 22-month survival when compared to those who did not develop AKI (79.2 months vs. 95.2 months, *p* = 0.049) (Table 6). Multivariate Cox survival analysis including age, CRPC, and AKI was performed. A single AKI episode was identified as an independent risk factor mortality when compared with the absence of AKI during abiraterone treatment [HR 3.044 (95% CI 1.001–9.260); *p* = 0.05] (Table 7)). Figure 1 shows the survival curves of patients with and without AKI during abiraterone treatment.

## 4. Discussion

In our study, 79 patients with CRPC and/or metastatic prostate cancer under abiraterone treatment were included and followed up for 17 months. Of those, 63.3% developed a renal adverse event: 36.7% electrolyte imbalance, 30.4% AKI, 25.5% new onset or worsening HTN, and 16.5% volume overload. Metastatic disease and previous HTN were identified as independent risk factors for the development of renal adverse events (metastatic disease: OR 13.335, 95% CI 1.418–124.444, *p* < 0.0235; previous HTN: OR 3.336; 95% CI 1.091–10.206; *p* < 0.0347). At the end of follow-up, 18.9% patients had died, and AKI was identified as an independent risk factor for mortality in this cohort [HR 3.044 (95% CI 1.001–9.260); *p* = 0.05].

Abiraterone is a selective inhibitor of the enzyme CYP17, which is responsible for converting pregnenolone and progesterone into testosterone and cortisol, respectively. By inhibiting CYP17 activity, abiraterone reduces the synthesis of both testosterone and cortisol. The resulting decrease in these hormones activates a negative feedback mechanism that increases the secretion of ACTH, subsequently leading to enhanced aldosterone production. This may result in secondary hyperaldosteronism, manifesting as edema, hypertension, electrolyte imbalances, fluid retention, and potentially acute renal failure. Therefore, to prevent excessive ACTH stimulation, abiraterone is co-administered with corticosteroids such as prednisone or prednisolone.

In the initial studies of abiraterone without concomitant corticosteroid therapy, adverse events related to hyperaldosteronism were reported [arterial hypertension (17–40%), volume overload (5–31%), and hypokalemia (48–88%)] [11,12]. For this reason, abiraterone is currently administered together with glucocorticoids. De Bono et al. compared adverse events of abiraterone–prednisone versus placebo–prednisone [13]. They observed that patients treated with abiraterone presented more symptoms related to hyperaldosteronism compared with those receiving placebo (55% vs. 41%, *p* < 0.001), with higher rates of hypokalemia (17% vs. 8%, *p* < 0.001) and volume overload (31% vs. 22%; *p* = 0.04). Similar findings were reported in the COU-AA-302 trial [14]: patients on abiraterone presented more hypertension (24% vs. 14%), hypokalemia (17% vs. 13%), and volume overload (31% vs. 24%) compared with placebo.

Our results are more consistent with those reported in the early studies of abiraterone monotherapy despite prednisone was used in all patients included in our study, mainly regarding to HTN (25.52%) and volume overload (16.45%), although we observed a lower incidence of hypokalemia (31.65%). Concomitant glucocorticoid therapy reduces, but does not eliminate, the risk of abiraterone-induced hyperaldosteronism, which is reflected in our results. Tatsuzawa et al. [15] demonstrated that the use of dexamethasone or prednisolone as concomitant therapy did not result in significant differences in potassium levels after treatment initiation. Bretagne M. et al. [16] reported a reporting odds ratio of approximately 2 for heart failure in abiraterone users compared to enzalutamide, based on three pharmacovigilance databases (French, European and global). They showed that patients with heart failure or tachyarrhythmias had higher rates of hypokalemia (26.3% vs. 14.6%), hypertension (15.8% vs. 5.6%), and volume overload (36.8% vs. 5.6%; *p* < 0.0001) compared with those experiencing other adverse events. Furthermore, they suggested that preexisting HTN increases the risk of tachyarrhythmias or heart failure.

In our cohort, 30.38% of patients treated with abiraterone developed AKI, with a median onset of 18 months after treatment initiation. Our findings are higher than those reported in other studies, such as Scailteux et al. [7], who described an incidence of 2.2%. However, that study defined AKI based on diagnostic coding from medical records, which may have underestimated the incidence. In contrast, in our study, AKI was defined as an increase in serum creatinine > 0.3 mg/dL above baseline renal function. Pivotal clinical trials and meta-analyses do not identify acute kidney injury as a frequent adverse event when abiraterone is used, but pharmacovigilance data suggest caution, particularly in patients with renal comorbidities or advanced age [17,18].

No studies have previously addressed the incidence of CKD following AKI during abiraterone therapy. In our study, 50% of patients who developed AKI progressed to CKD, and among them, one in four developed moderate-to-advanced CKD (stage IV). These findings highlight the potential severity of AKI as a precipitating factor for chronic renal deterioration in this population.

As far as we know, this is the first study to establish that AKI represents a risk factor for mortality in patients treated with abiraterone for prostate cancer. Schoen et al. [19] demonstrated that US veterans receiving abiraterone or enzalutamide with eGFR < 30 mL/min (CKD stage IIIB-IV) died more frequently than others with eGFR > 30 mL/min (aHR 1.08; 95% CI 0.93–1.25), with no statistical significance. They also demonstrated that patients with cardiovascular disease or diabetes who received enzalutamide had 13% decreased mortality (aHR 0.87, 95% CI 0.81–0.93) compared to those who received abiraterone.

Although our study is the first that describes a single episode of AKI as a risk factor for mortality, it has some limitations. We acknowledge its retrospective character and short follow-up. It is a single-center study, and our center is a reference center, a fact that could reflect a more severe scenario. Furthermore, several confidence intervals are extremely wide, what could limit the statistical power.

In conclusion, we provide novel evidence showing that a single episode of AKI is an independent risk factor for mortality in patients treated with abiraterone. HTN was identified as a risk factor for AKI in our study, where the overall incidence of AKI was 30.38%. Our results suggest that further growth of the subspecialty of Onconephrology is needed to improve the diagnosis and management of renal and electrolyte-related adverse events in patients receiving oncological therapies.

## Figures and Tables

**Table 1:** Baseline characteristics and univariate analysis comparing patients who presented renal adverse events and patients who did not.

Clinical and Demographic Parameters	Cohort (n = 79)	Renal Event (n = 50)	No Renal Event (n = 29)	*p* Value
Age (years), mean (SD)	76.05 ± 9.5	76.80 ± 9.09	74.76 ± 10.17	0.3601
CRPC (yes), n (%)	56 (70.9)	37 (74)	13 (44.82)	0.4237
Metastatic disease (yes), n (%)	71 (89.87)	49 (98)	22 (75.86)	**0.0017**
Baseline creatinine (mg/dL), median (IQR)	0.9 (0.8–1.05)	0.90 (0.80–1.10)	0.90 (0.80–1.00)	0.8648
CKD (yes), n (%)	17 (21.5)	11 (22)	6 (20.7)	0.8194
Nephrectomy (yes), n (%)	1 (1.26)	1 (2)	0 (0)	0.4434
Urinary diversion (yes), n (%)	4 (5.06)	4 (8)	0 (0)	0.1180
Previous AKI (yes), n (%)	17 (21.5)	15 (30)	2 (6.89)	**0.0110**
Use of diuretics (yes), n (%)	18 (22.78)	12 (24)	6 (20.7)	0.7353
Diabetes mellitus (yes), n (%)	7 (8.86)	5 (10)	2 (6.9)	0.6399
Pre-existing HTN (yes), n (%)	44 (55.7)	32 (64)	12 (41.38)	0.0511
Sodium value (mmol/L), median (IQR)	139 (138–141)	140 (138–141)	139 (141–147)	0.4894
Potassium value (mmol/L), median (IQR)	4.4 (4.2–4.4)	4.4 (4.20–4.6)	4.4 (4.20–4.6)	0.9160

AKI: acute kidney injury; CKD: chronic kidney disease; CRPC: castration-resistant prostate cancer; eGFR: estimated glomerular filtration rate; HTN: hypertension; IQR: interquartile range; SD: standard deviation.

**Table 2:** Independent risk factors for renal adverse events in patients under abiraterone treatment. Logistic regression model.

Characteristics	Odds Ratio	95% CI	*p*-Value
Baseline creatinine (mg/dL)	0.185	0.013–6.412	0.1735
History of HTN (yes)	3.336	1.091–10.206	**0.0347**
Previous AKI (yes)	5.969	0.986–36.149	0.0518
Metastatic cancer (yes)	13.335	1.418–124.444	**0.0235**

AKI: acute kidney injury; HTN: hypertension.

**Table 3:** Univariate analysis comparing characteristics of patients who presented AKI and those who did not.

	AKI Yes n = 24	AKI Event No n = 55	*p*
Age (years). mean ± SD	77.25 ±	74.98 ± 10.37	0.3387
Castration resistant cancer (yes)	20	32	0.0713
Metastatic cancer. n (%)	23	45	0.2914
Hypertension. n (%)	18	24	**0.0230**
Diabetes. n (%)	2	5	0.8382
Diuretics. n (%)	6	12	0.8894
Baseline creatinine (mg/dL). median (IQR)	1 (0.8–1.2)	0.8 (0.8.1.00)	**0.0274**
CKD. n (%)	8	7	0.1175
Previous AKI. n (%)	8	8	**0.0376**
Sodium value (mmol/L). median (IQR)	140 (138–141.5)	139 (138–141)	0.6959
Potassium value (mmol/L). median (IQR)	4.4 (4.2–4.7)	4.4 (4.125–4.6)	0.5102
Nephrectomy. n (%)	0	1	0.4898
Urinary diversion. n (%)	2	2	0.4277

**Table 4:** Univariate analysis comparing characteristics of patients who died and patients who survived at the end of follow-up.

Characteristics	Death (n = 15)	Survival (n= 64)	*p*-Value
Age (years). mean (SD)	76.89 ± 9.14	75.75 ± 9.66	0.6847
CRPC (yes). n (%)	14 (93.3)	41 (64.1)	**0.0310**
Metastatic cancer (yes). n (%)	15 (100)	55 (85.9)	0.1452
Pre-existing HTN (yes). n (%)	9 (60)	35 (54.7)	0.7551
Diabetes mellitus (yes). n (%)	1 (6.7)	6 (9.4)	0.7279
CKD (yes). n (%)	4 (26.7)	13 (20.3)	0.5375
Renal event (yes). n (%)	11 (73.3)	39 (60.9)	0.4070
AKI (yes). n (%)	9 (60)	15 (23.44)	**0.041**
De novo or worsening hypertension (yes). n (%)	4 (26.7)	13 (20.3)	0.5375
Electrolyte imbalance (yes). n (%)	8 (53.3)	21 (32.8)	0.1385
Volume overload (yes). n (%)	5 (33.3)	19 (29.7)	0.6848

AKI: acute kidney injury; CKD: chronic kidney disease; CRPC: castration-resistant prostate cancer; HTN: hypertension; SD: standard deviation.

**Table 5:** Independent risks factors for AKI in patients under abiraterone treatment. Logistic regression model.

	Odds Ratio	95% CI	*p*-Value
History of HTN	3.946	1.127–13.818	**0.0318**
Baseline creatinine (mg/dL)	4.646	0.340–63.448	0.2494
Previous AKI	1.818	0.453–7.287	0.3989

AKI: acute kidney injury; HTN: hypertension.

**Table 6:** Cumulative probability of survival according to Kaplan–Meier analysis at 22 months after abiraterone initiation and univariate Cox analysis for mortality.

		22-Months Cumulative Probability of Survival% (SE)	HR (95% CI)	*p*
AKI	yes	79.2 (8.3)	3.3 (0.108–0.999)	**0.049**
	no	92.5 (3.1)	1	
Age ≥ 76 years old	yes	81.5 (6.5)	1.95 (0.66–5.77)	0.229
	no	88.4 (5.5)	1	
CRPC	yes	78.9 (5.8)	5.45 (0.72–41.57)	0.102
	no	100	1	
Metastatic cancer	yes	83.3 (4.7)	23.73 (0.004–156624.07)	0.480
	no	100	1	
Pre-existing HTN	Yes	84.1 (6.7)	1.21 (0.43–3.39)	0.724
	no	85.3 (6.7)	1	
Diabetes mellitus	Yes	83.8 (5.1)	1.15 (0.32–4.17)	0.824
	No	88.2 (7.8)	1	
CKD	Yes	82.4 (9.2)	1.67 (0.51–5.43)	0.393
	No	85.0 (5.1)		
Renal event	Yes	71.7 (1.67)	1.20 (0.35–4.17)	0.766
	No	85.8 (5.0)	1	
Denovo/worsening HTN	Yes	84.2 (5.4)	1.02 (0.32–3.29)	0.976
	No	88.2 (7.8)	1	
Electrolyte imbalance	Yes	82.8 (7.0)	1.55 (0.53–4.54)	0.424
	No	85.6 (6.3)	1	
Volume overload	Yes	82.3 (6.0)	1.27 (0.41–3.84)	0.684
	No	85.1 (8.2)	1	

AKI: acute kidney injury; CKD: chronic kidney disease; CRPC: castration-resistant prostate cancer; HR: hazard ratio; HTN: hypertension; SE: standard error.

**Table 7:** Independent risk factors for mortality. Multivariate Cox analysis.

	HR (95% CI) for Mortality	*p*
Age (years)	0.994 (0.932–0.1059)	0.843
CRPC (yes)	4.000 (0.510–31.363)	0.105
AKI (yes)	3.044 (1.001–9.260)	**0.05**

AKI: acute kidney injury; CRPC: castration-resistant prostate cancer; HR: hazard ratio.

## Data Availability

The data that support the findings of this study are available from the corresponding author upon reasonable request. These results have been previously presented at the Annual Meeting of the Madrid Society of Nephrology (SOMANE) 2025 (Segovia, Spain, June 2025), at the 62nd Congress of the European Renal Association (Vienna, Austria, June 2025), and at the 55th Congress of the Spanish Society of Nephrology (Oviedo, Spain, October 2025). The Ethics Committee of Hospital Clínico San Carlos approved the publication of this article after reviewing and analyzing the manuscript (internal ID 25-044).

## Abbreviations

The following abbreviations are used in this manuscript:

ACTH	adrenocorticotropic hormone
ADT	androgen deprivation therapy
aHR	adjusted hazard ratio
AKI	acute kidney injury
CI	confidence interval
CKD	chronic kidney disease
CRPC	castration-resistant prostate cancer
CYP17	17α-hydroxylase/C17,20-lyase
eGFR	estimated glomerular filtration rate
HR	hazard ratio
HTN	hypertension
IQR	interquartile range
KDIGO	Kidney Disease: Improving Global Outcomes
mCRPC	metastatic castration-resistant prostate cancer
mHSPC	metastatic hormone-sensitive prostate cancer
SD	standard deviation
OATP1B1	organic anion transporting polypeptide 1B1
OR	Odds ratio
